# Impact of Early Consumption of High-Fat Diet on the Mesolimbic Dopaminergic System

**DOI:** 10.1523/ENEURO.0120-17.2017

**Published:** 2017-06-01

**Authors:** F. Naneix, F. Tantot, C. Glangetas, J. Kaufling, Y. Janthakhin, C. Boitard, V. De Smedt-Peyrusse, J. R. Pape, S. Vancassel, P. Trifilieff, F. Georges, E. Coutureau, G. Ferreira

**Affiliations:** 1 Université de Bordeaux, 33077 Bordeaux, France; 2 CNRS, Institut de Neurosciences Cognitives et Intégratives d'Aquitaine, UMR 5287, 33077 Bordeaux, France; 3 INRA, Nutrition et Neurobiologie Intégrée, UMR 1286, 33077 Bordeaux, France; 4 CNRS, Institut des Maladies Neurodégénératives, UMR 5293, 33077 Bordeaux, France

**Keywords:** adolescence, amphetamine, dopamine, high-fat diet, nucleus accumbens, sensitization

## Abstract

Increasing evidence suggest that consumption of high-fat diet (HFD) can impact the maturation of brain circuits, such as during adolescence, which could account for behavioral alterations associated with obesity. In the present study, we used behavioral sensitization to amphetamine to investigate the effect of periadolescent HFD exposure (pHFD) in rats on the functionality of the dopamine (DA) system, a central actor in food reward processing. pHFD does not affect responding to an acute injection, however, a single exposure to amphetamine is sufficient to induce locomotor sensitization in pHFD rats. This is paralleled by rapid neurobiological adaptations within the DA system. In pHFD-exposed animals, a single amphetamine exposure induces an increase in bursting activity of DA cells in the ventral tegmental area (VTA) as well as higher DA release and greater expression of (tyrosine hydroxylase, TH) in the nucleus accumbens (NAc). Post-synaptically, pHFD animals display an increase in NAc D2 receptors and c-Fos expression after amphetamine injection. These findings highlight the vulnerability of DA system to the consumption of HFD during adolescence that may support deficits in reward-related processes observed in obesity.

## Visual Abstract

**Figure:**
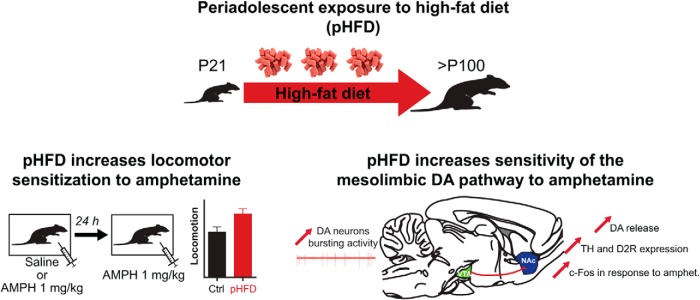

## Significance Statement

Consumption of obesogenic diet might impact the development of the reward system, leading to cognitive and behavioral alterations associated with obesity. This study investigates the effects of high-fat diet (HFD) consumption, from childhood to adulthood, on the functionality of mesolimbic dopamine (DA) system using sensitization to amphetamine. We show that a single exposure to amphetamine is sufficient to induce behavioral sensitization in HFD-exposed animals. This is associated with sensitization of the DA mesolimbic pathway, with higher bursting activity of DA neurons and enhanced DA release, greater expression of tyrosine hydroxylase (TH), D2 receptors and c-Fos levels in the NAc. This study demonstrates that early exposure to obesogenic diet consumption alters the sensitivity of DA system that may lead to reward-related disorders.

## Introduction

Adolescence is a critical period of life characterized by major cognitive and neurobiological changes (Spear, 2000), making it a window of vulnerability to pathologic development (Andersen, 2003; Adriani and Laviola, 2004; Paus et al., 2008; Reichelt, 2016). Adolescents are particularly sensitive to rewards and often increase their consumption of palatable foods such as high-fat diet (HFD; Crews et al., 2007; Ogden et al., 2012), which could lead to obesity. The long-term consequences of chronic consumption of palatable foods during adolescence remain unclear but might lead to alterations of the brain reward system that have been associated with obesity and feeding disorders (Berthoud and Morrison, 2008; Kenny, 2011; Volkow et al., 2011; Reichelt, 2016).

The dopamine (DA) system plays a central role in incentive processes for natural and artificial rewards (Berridge and Robinson, 1998; Norgren et al., 2006; Wise, 2006; Fulton, 2010). It has been proposed that consumption of palatable foods, by increasing DA release in the nucleus accumbens (NAc; Norgren et al., 2006; Wise, 2006), could reinforce associations between environmental cues or actions with the food (Volkow et al., 2011). In both humans and rodents, numerous studies have reported enhancement of incentive processes in obese subjects or after the consumption of obesogenic diet in adults (Wang et al., 2009; Johnson and Kenny, 2010; Volkow et al., 2011; Wang et al., 2011; Robinson et al., 2015). The impact of obesogenic diet on the DA system remains unclear (Kenny, 2011; Décarie-Spain et al., 2015) and previous work reported either blunted DA activity (Davis et al., 2008; Johnson and Kenny, 2010) or increased DA response (McGuire et al., 2011; Volkow et al., 2011; Baladi et al., 2015; Fordahl et al., 2016) in response to food and drug that may both drive increased reward-seeking behaviors.

The DA system displays delayed maturation that takes place during adolescence, making it vulnerable to environmental influences (Spear, 2000; Andersen, 2003; Naneix et al., 2012; Naneix et al., 2013). Interestingly, sucrose consumption during adolescence leads to long-lasting deficits of reward processing (Frazier et al., 2008; Vendruscolo et al., 2010; Naneix et al., 2016) and HFD consumption in adolescent but not adult rats increases locomotor sensitivity to psychostimulants (Baladi et al., 2015; Fordahl et al., 2016), suggesting a particular impact of high-energy diet consumed during adolescence on the DA system (Reichelt, 2016).

In the present study, we investigated the effects of periadolescent HFD consumption (pHFD; from weaning to adulhood; Boitard et al., 2014; Boitard et al., 2015; Labouesse et al., 2016; Tantot et al., 2016) on the functionality of the mesolimbic DA system, i.e., the ventral tegmental area (VTA)-NAc pathway. For this purpose we performed behavioral sensitization to amphetamine, classically used to investigate changes in VTA-NAc DA transmission induced by repeated exposure to drugs of abuse (Robinson and Berridge, 1993; Vanderschuren and Kalivas, 2000; Steketee and Kalivas, 2011). to probe the discrete changes induced by pHFD within the DA system, but to overcome the long-lasting changes associated with the development of tolerance and dependence, we used a two-injection amphetamine protocol (Vanderschuren et al., 1999; Valjent et al., 2005; Chinen et al., 2006; Valjent et al., 2010). We demonstrate that pHFD potentiates locomotor sensitization induced by a single exposure to amphetamine. Using a multi-level approach, we then show that this behavioral effect is associated with rapid adaptations of the DA mesolimbic pathway, encompassing an increased activity of DA cells in the VTA and an enhancement of DA release, expression of DA synthesis enzyme (tyrosine hydroxylase, TH), D2 receptors and c-Fos levels in the NAc. Taken together, these data reveal the vulnerability of the DA system to HFD consumption during adolescence that may support long-term alterations of reward processing and feeding.

## Materials and Methods

### Subjects and diet

Male Long-Evans rats (RRID:RGD_60991; Janvier) were received at the age of three weeks and were housed by two in polycarbonate cages (48 × 26 × 21 cm) in a temperature (22 ± 1°C) and humidity-controlled room maintained under a normal 12/12 h light/dark cycle (lights on at 7 A.M.). The experiments took place in the light phase of the cycle. Food and water were provided ad libitum. Diets consisted in either control diet (CD, *n* = 101) providing 3.1 kcal/g [consisting of 3% lipids (8% kcal), 16% proteins (19% kcal), and 60% carbohydrate (73% kcal); A04, SAFE] or a HFD (*n* = 119) providing 4.7 kcal/g [consisting of 24% lipids (45% kcal), mostly saturated fat from lard, 24% proteins (20% kcal), and 41% carbohydrates (35% kcal); D12451, Research Diets]. Rats were exposed to CD or HFD for three months from weaning (postnatal day 21) to adulthood (postnatal days 110-120). All experiments took place during adulthood. HFD consumption exceeded adolescence which is usually considered to be approximately postnatal days 30-60 in male rats (Spear, 2000; Andersen, 2003; Schneider, 2013). That is the reason why we used the term pHFD. Previous studies have shown a more pronounced cognitive and neurobiological impact of pHFD compared with similar HFD exposure starting at adulthood (Boitard et al., 2014; Boitard et al., 2015; Labouesse et al., 2016). The fact that similar HFD exposure at adulthood did not lead to similar impact discarded any acute influence of HFD intake on behavior. As we recently reported (see Table 1 in Tantot et al., 2016), male Long-Evans rats exposed to pHFD were 10% heavier than their respective controls (373 vs 336 g) and showed significant increased levels of leptin (+100%) and to a lesser extent of insulin and cholesterol (+30%) but not triglycerides.

**Table 1.** Effect of pHFD on the expression of DA markers in the NAc and the VTA of nonsensitized rats

	NAc			VTA		
	Mean ± SEM	Student’s *t* test	*p* value	Mean ± SEM	Student’s *t* test	*p* value
TH	CD: 100 ± 8	*t*_(19)_ = 0.4	*p* = 0.7; NS	CD: 100 ± 13	*t*_(19)_ = 2.1	*p* < 0.05; *
	HFD: 95 ± 12			HFD: 63 ± 11		
DAT	CD: 100 ± 10	*t*_(19)_ = 0.004	*p* = 0.9; NS	CD: 100 ± 12	*t*_(19)_ = 0.01	*p* < 0.9; NS
	HFD: 100 ± 10			HFD: 100 ± 10		
D1R	CD: 100 ± 11	*t*_(18)_ = 0.4	*p* = 0.6; NS			
	HFD: 106 ± 7					
D2R	CD: 100 ± 11	*t*_(18)_ = 0.2	*p* = 0.8; NS			
	HFD: 104 ± 12					

Expression levels of TH, DAT and DA receptors (D1R and D2R). All data are expressed as mean ± SEM and in % of CD group. NS, nonsignificant; *p < 0.05 diet effect.

Experiments were conducted in agreement with the French (council directive 2013-118, February 1, 2013) and international legislation (directive 2010-63, September 22, 2010, European Community) and were approved (agreement number 5012047-A) by the Bordeaux Ethics Committee (CNREEA no. 50).

### Locomotor Activity and sensitization

Twenty-four hours before locomotor activity testing, rats received an injection of either saline (no sensitization) or amphetamine (1 mg/kg, i.p., dissolved in 0.9% saline at 1 mg/ml, Sigma Aldrich; sensitization). The day of testing, all rats first received an injection of saline and their spontaneous locomotor activity was measured during 60 min using individual cages (23 × 36 × 19 cm, Imetronic) equipped with two grids of photobeam sensors (3 × 37 × 3 cm located at 3 and 9 cm above the floor). Then, rats were injected with saline (saline group; CD *n* = 12, pHFD *n* = 16) or amphetamine (1 mg/kg; no sensitization CD *n* = 7 and pHFD *n* = 9; sensitization CD *n* = 11; pHFD *n* = 17) and were then recorded for an additional 60 min.

### *In vivo* recording of VTA-DA neurons

Twenty-four hours after either saline 0.9% (CD *n* = 5, pHFD *n* = 4) or amphetamine (1 mg/kg; CD *n* = 5, pHFD *n* = 4) injection in their home cage, rats were anesthetized with isoflurane. A glass micropipette (tip diameter, 2–3 μm; 4–6 MΩ) filled with a 2% pontamine sky blue solution in 0.5 M sodium acetate was lowered into the VTA (AP −5.3 mm, ML ±0.7 mm, DV −7.5 mm from dura; Paxinos and Watson, 1998) as previously described (Georges and Aston-Jones, 2002). VTA-DA neurons were identified according to well established electrophysiological features (Grace and Bunney, 1983; Ungless and Grace, 2012) which included (1) action potential with biphasic or triphasic wave form >2.5 ms in duration, (2) slow spontaneous firing rate (<10 Hz), (3) single and burst spontaneous firing patterns (characterized by spike–amplitude decrement). Signals were amplified and filtered (0.1–5 kHz bandpass) using conventional electronics. Single-neuron spikes were discriminated and digital pulses were led to a computer for on-line data collection with the use of a laboratory interface and software (CED 1401, SPIKE 2; Cambridge Electronic Design; RRID:SCR_000903).

Four parameters for VTA-DA neurons were analyzed: the basal firing rate, the bursting rate (number of burst events per second), the percentage of spikes that occurred in bursts (%SIB) and the burst size (number of spikes per burst). DA neurons were also classified according to their modes of firing pattern based of firing rate and %SIB (Mameli-Engvall et al., 2006): (1) low-frequency and low-burst firing (firing rate <5 Hz and %SIB <20%), (2) low-frequency and high-burst firing (firing rate < 5 Hz and %SIB > 20%), (3) high-frequency and low-burst (firing rate > 5 Hz and %SIB <40%), and (4) high-frequency and high-burst firing (firing rate > 5 Hz and %SIB > 40%). Electrophysiological recording sites were confirmed by iontophoretic deposit of pontamine sky blue dye.

### Microdialysis

Twenty-four hours after either saline 0.9% (CD *n* = 9, pHFD *n* = 11) or amphetamine (1 mg/kg; CD *n* = 11, pHFD *n* = 10) injection in their home cage, rats were anesthetized with urethane (1.5 g/kg, i.p.). A unilateral microdialysis probe (CMA 12 Elite, Phymep) was stereotaxically inserted in the NAc: AP +1.7 mm, ML ±1.1 mm, DV -7.5 mm from the dura (Paxinos and Watson, 1998). Artificial cerebro-spinal fluid (149 mMNaCl, 1 mM NaH_2_PO_4_, 3 mMKCl, 1 mM MgCl_2_, and 1.4 mM CaCl_2_, pH 7.4) was pumped through the probe during 1 h for equilibration (2.5 µl/min). Samples were collected every 20 min for 1 h before and 2 h after amphetamine injection (1 mg/kg) and were stored at -80°C after addition of 5 µl of HCl. After the experiment, rats were sacrificed and brains were removed. Coronal sections (50 µm) were collected and stained with cresyl violet to determine probe placement. Eleven rats were removed after histologic control. The final group size was: no sensitization CD *n* = 6 and pHFD *n* = 8; sensitization CD *n* = 9 and pHFD *n* = 8.

The dialysate samples (50 μl) were injected into a high-performance liquid chromatography equipped with a 5-µm C18, 3 × 100 mm silica column (ACE, AIT) and a DECADE II detector (Antec Leyden) to quantify DA. The mobile phase, consisting of 0.1 M citric acid, 0.1 M dibutylamine, 0.5 mM octanesulfonic acid, and 0.1 mM EDTA, pH 3.5, was pumped at 0.3 mL/min (Dionex SA) through oxidation potential of the electrochemical detector (Decade 2, Antec) set at 600 mV. Signals were recorded and quantified with Chromeleon chromatography data system (Dionex SA). DA levels concentrations were calculated against a daily injected standard.

### DA and metabolites tissue levels

CD (*n* = 10) or HFD (*n* = 17) naïve rats were sacrificed at adulthood and brains were quickly removed. NAc was dissected and snap-frozen before analysis. Tissue levels of DA and metabolites (DOPAC) were quantified by HPLC-ED as previously described (Parrot et al., 2011).

### c-Fos immunostaining

Twenty-four hours after amphetamine sensitization, rats received a second injection of either saline 0.9% (CD *n* = 5, pHFD *n* = 5) or amphetamine (1 mg/kg; CD *n* = 4; pHFD *n* = 5) in their home cage. Ninety minutes later, rats were sacrificed with an overdose of pentobarbital sodium and perfused transcardially with 0.1 M PBS (pH 7.4), followed by 4% paraformaldehyde in PBS. Brains were postfixed overnight in 4% paraformaldehyde and then transferred in 30% sucrose PBS solution for 48 h. Finally, brains were frozen in isopentane and stored at -80°C. Coronal sections (40 µm) were generated on a cryostat and incubated in blocking solution (PBS, Triton X-100 0.3%, BSA 3%) for 45 min, then with primary anti c-Fos antibody (1:1000 in blocking solution; Santa Cruz Biotechnology; RRID:AB_2106783) for 24 h at 4°C. After rinses, sections were then incubated in PBS-H_2_O_2_ 0.3% for 30 min, rinsed, and incubated with secondary antibody (biotinylated donkey anti-rabbit 1:2000; Jackson ImmunoResearch; RRID:AB_2340593) for 2 h at room temperature. They were then incubated with avidin-biotin-peroxydase complex (1:1000; Vector Laboratories) for 1 h at room temperature. The staining was revealed after 10-min incubation in a mix of diaminobenzidine, ammonium chloride, ammonium sulfate, sodium acetate, glucose and glucose oxydase. The reaction was stopped by incubation in sodium acetate (2 × 10 min). c-Fos labeling was quantified bilaterally on three sections spaced 240 µm apart and chosen to cover the NAc and the medial prefrontal cortex according to the Paxinos and Watson atlas (Paxinos and Watson, 1998). Each section was photographed using Nikon-ACT-1 software, and labeled cells were counted with ImageJ software (RRID:SCR_003070) on a surface representing 1 mm^2^.

### Western blotting

Twenty-four hours after either saline 0.9% (CD *n* = 11, pHFD *n* = 10) or amphetamine (1 mg/kg; (CD *n* = 11, pHFD *n* = 11) injection in their home cage, rats were killed and brain regions were manually dissected, frozen in dry ice and stored at −80°C before analysis. For the analysis of DA receptors, two pHFD rats were removed due to the absence of signal (no sensitization *n* = 9; sensitization *n* = 10). Tissue samples were lysed in 300 µl of extraction buffer respectively containing 50 mM Tris, 2% SDS, 5 M urea, and phosphatase/protease inhibitor cocktail (Thermo Fisher) and were sonicated (amplitude 80%, 4 × 1 s) on ice. Protein contents were determined by the MicroBCAssay (Uptima, Interchim) according to the manufacturer’s protocol. For DA transporter (DAT), TH, and D1R detection, 5 µg of protein, diluted in 2× Laemmli buffer, were heated for 5 min at 75°C and loaded on a 4–15% polyacrylamide gradient gel (D1556, Bio-Rad). For D2R, 10 µg of protein diluted in 2× Laemmli buffer were loaded on 12% acrylamide gel. Proteins were transferred on nitrocellulose membrane (Protran Premium 0.2 µm, GE Healthcare) using a Miniprotean system (Bio-Rad). Membranes were saturated with 5% fat-free dry milk in TBS-Tween 0.1% for 1h at room temperature and probed overnight at 4°C with primary antibodies: DAT (1:1000, AB2231, Millipore; RRID:AB_1586991), D1R (1:1000, D2944, Sigma Aldrich; RRID:AB_1840787), TH (1:5000, MAB318, Millipore; RRID:AB_2313764) and D2R (kindly provided by Prof. J. Javitch, Columbia University). Anti-β-actin (1:2500, Biolegend; RRID:AB_315945) or anti-GAPDH (1:5000, Cell Signaling Technolology; RRID:AB_10622025) antibodies were used against internal markers to normalize protein expression. Primary antibodies were detected with appropriated donkey horseradish peroxidase-conjugated secondary antibodies (1:5000, Jackson ImmunoResearch). The blots were developed by using Supersignal Westdura (Thermo Fisher). Specific protein signals were quantified by measuring chemiluminescence with Chemidoc Detection System and Image Lab Software (Bio-Rad).

### Statistical analysis

Statistical analyses were conducted using GraphPad Prism 6 (RRID:SCR_002798). Data were analyzed using two-tailed Student’s *t* test or ANOVA with or without repeated measures when appropriate, followed by Bonferroni's *post hoc* tests. Normality was checked with the Shapiro-Wilk test. As electrophysiological parameters did not follow a normal law (except firing rate), nonparametric tests were used (Kruskall-Wallis and Mann–Whitney *U* tests). The α risk for rejection of the null hypothesis was fixed at 0.05.

## Results

### pHFD increases amphetamine-induced locomotor sensitization

**Figure 1.**
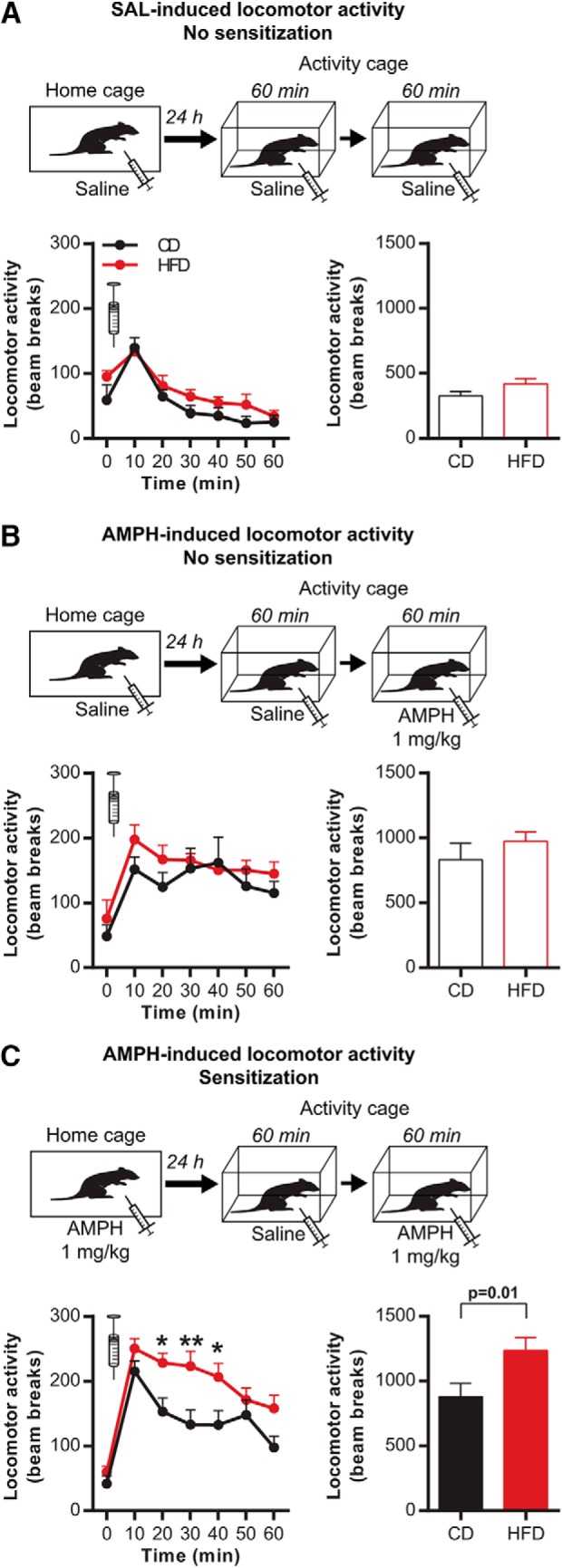
pHFD increases amphetamine-induced locomotor sensitization. ***A***, Locomotor activity (photobeam counts) in response to saline was not affected by pHFD in nonsensitized animals (CD *n* = 12; pHFD *n* = 16). ***B***, Locomotor activity in response to amphetamine was not affected by pHFD in nonsensitized animals (CD *n* = 7; pHFD *n* = 9). ***C***, pHFD diet increased locomotor activity in response to amphetamine in sensitized animals (CD *n* = 11; pHFD *n* = 17). Left panels, Locomotor activity every 10 min. Right panels, Cumulative locomotor activity during 60 min. Syringes represent the time of amphetamine injection. Data are expressed as mean + SEM. *p < 0.05, **p < 0.01 diet effect.

The behavioral effect of pHFD on the functionality of the mesolimbic DA system was first investigated using locomotor response and sensitization to amphetamine. CD or pHFD-exposed rats received a first injection of saline (no sensitization) or amphetamine (1 mg/kg; sensitization; Fig. 1). Twenty-four hours later, their locomotor activity was first measured during 60 min in response to an injection of saline. pHFD did not affect basal locomotor activity of nonsensitized (CD: 792 ± 71/pHFD: 887 ± 57; *t*_(30)_ = 1.1, *p* = 0.2) and sensitized rats (CD: 910 ± 61/pHFD: 767 ± 54; *t*_(42)_ = 1.7, *p* = 0.09). In accordance with these results, nonsensitized CD and pHFD groups responded similarly to a second injection of saline (diet: *F*_(1,26)_ = 3.5, *p* = 0.07; time: *F*_(6156)_ = 19.9, *p* < 0.001; interaction: *F*_(6156)_ = 0.7, *p* = 0.6; Fig. 1*A*) or to a first injection of amphetamine, which similarly increased locomotor activity in CD and pHFD rats (diet: *F*_(1,14)_ = 1.2, *p* = 0.3; time: *F*_(6,84)_ = 8.9, *p* < 0.001; interaction: *F*_(6,84)_ = 0.63, *p* = 0.7; Fig. 1*B*). Interestingly, pHFD sensitized rats displayed higher locomotor activity in response to a second injection of amphetamine compared with CD rats (diet: *F*_(1,42)_ = 6.2, *p* = 0.01; time: *F*_(6252)_ = 41.4, *p* < 0.001; interaction: *F*_(6252)_ = 2.6, *p* < 0.1; Fig. 1*C*). Comparison of sensitized and nonsensitized animals indicated a tendency toward a more sustained locomotor activity in pHFD sensitized rats (time × sensitization interaction: *F*_(6198)_ = 1.9, *p* = 0.08; Fig. 1*B*,*C*) which was not observed in CD rats. These results demonstrated that pHFD rats sensitize faster than CD rats suggesting that periadolescent obesogenic diet might increase the reactivity of the DA system at adulthood.

### pHFD increases activity of DA mesolimbic pathway after amphetamine sensitization

**Figure 2.**
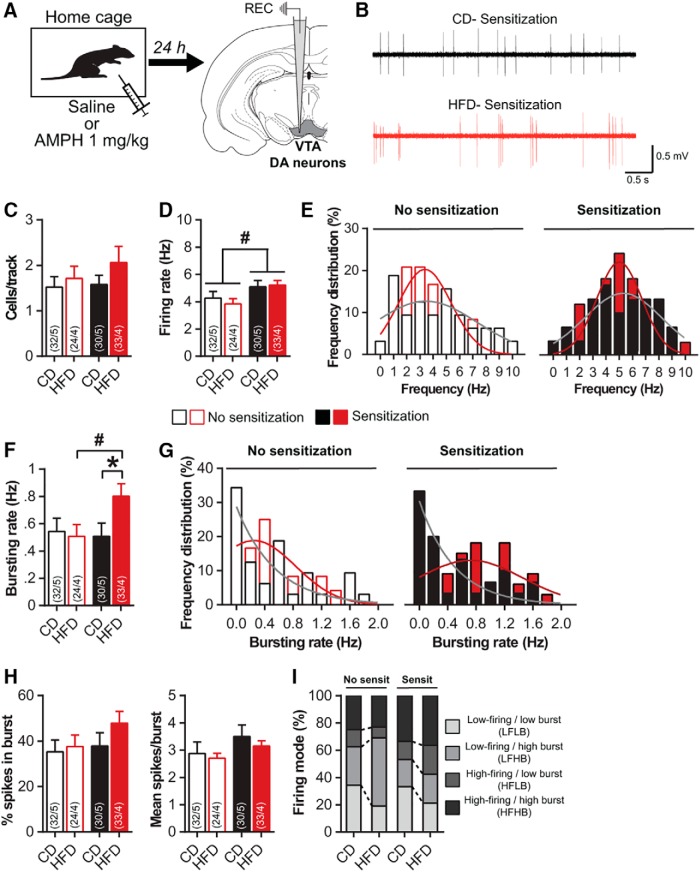
Amphetamine sensitization increases bursting activity of VTA-DA neurons after pHFD. ***A***, Experimental schematic: 24 h after saline or amphetamine injection, VTA-DA neurons were recorded on anesthetized rats (no sensitization: CD *n* = 5 and pHFD *n* = 4; sensitization CD *n* = 5 and pHFD *n* = 4). ***B***, Representative traces of VTA putative DA neurons for CD (top) and pHFD (bottom) groups 24 h after amphetamine injection. ***C***, Number of spontaneously active DA neurons in the VTA is not affected by pHFD or amphetamine sensitization. ***D***, Firing rate of VTA-DA neurons was not affected by pHFD but was increased by amphetamine sensitization. ***E***, Distribution of VTA-DA neurons firing rate was not affected by pHFD (Kolmogorov-Smirnov test; no sensitization: *D*_(56)_ = 0.19, *p* = 0.6; sensitization: *D*_(63)_ = 0.18, *p* = 0.7) but was right-shifted by amphetamine sensitization (CD: *D*_(62)_ = 0.21, *p* = 0.4; HFD: *D*_(57)_ = 0.36, *p* = 0.05). ***F***, Amphetamine sensitization increased the bursting rate of VTA-DA neurons only in pHFD rats. ***G***, Distribution of VTA-DA neurons bursting rate was shifted toward high frequency in pHFD sensitized animals (Kolmogorov-Smirnov test; no sensitization: *D*_(56)_ = 0.17, *p* = 0.8; sensitization: *D*_(63)_ = 0.35, *p* < 0.05; CD: *D*_(62)_ = 0.18, *p* = 0.6; HFD: *D*_(57)_ = 0.34, *p* = 0.07). ***H***, %SIB and burst size were not changed by pHFD or amphetamine sensitization. ***I***, Firing modes patterns are not affected by pHFD. Numbers in bars indicate the number of cells and rats. Solid lines in ***E***, ***G*** represent the best-fit distribution curve for the histogram data. Data are expressed as mean + SEM. *p < 0.05 diet effect; #*p* < 0.05 sensitization effect.

Because the VTA-NAc DA pathway plays a central role in locomotor response to drugs and behavioral sensitization (Vanderschuren and Kalivas, 2000; Ungless et al., 2001; Steketee and Kalivas, 2011), we next evaluated the impact of pHFD on the electrophysiological activity of VTA DA cells in anesthetized rats 24 h after saline or amphetamine administration (Fig. 2*A*,*B*). All groups exhibited a similar number of spontaneously active DA neurons per electrode track suggesting that neither pHFD nor amphetamine sensitization affected the population activity in the VTA (Kruskal--Wallis test; *K*_(3)_ = 1.6, *p* = 0.6; Fig. 2*C*). In nonsensitized rats, pHFD did not alter firing rate or bursting activity (Fig. 2*D-G*), demonstrating that HFD did not affect DA neurons functioning under basal conditions. Prior amphetamine sensitization significantly increased firing rate in both CD (+19%) and pHFD rats (+35%; diet *F*_(1115)_ = 0.1, *p* = 0.7; sensitization: *F*_(1115)_ = 6.4, *p* < 0.05; interaction: *F*_(1115)_ = 0.4, *p* = 0.5; Fig. 2*D-E*). Interestingly, sensitization induced a significant increase of bursting rate specifically in pHFD animals (Kruskal--Wallis test followed by Mann--Whitney *U* test; *K*_(3)_ = 7.1, *p* = 0.07; diet effect: no sensitization *U* = 372, *p* = 0.8; sensitization *U* = 344, *p* < 0.05; sensitization effect: CD *U* = 475, *p* = 0.9; HFD *U* = 260, *p* < 0.05; Fig. 2*F*,*G*), without affecting other parameters such as the %SIB or the number of spikes per burst (*K*_(3)_ = 3.3, *p* = 0.3 and *K*_(3)_ = 4.5, *p* = 0.2, respectively; Fig. 2*H*). Furthermore, despite the changes in both firing and bursting of DA neurons after sensitization, a more detailed analysis reported a similar distribution of firing patterns between CD and pHFD rats (χ^2^ test; no sensitization: χ^2^ = 3.9, *p* = 0.3; sensitization: χ^2^ = 1.5, *p* = 0.7; Fig. 2*I*). Taken together, these results demonstrate that pHFD sensitizes to amphetamine-induced increase in bursting activity of VTA DA cells.

**Figure 3.**
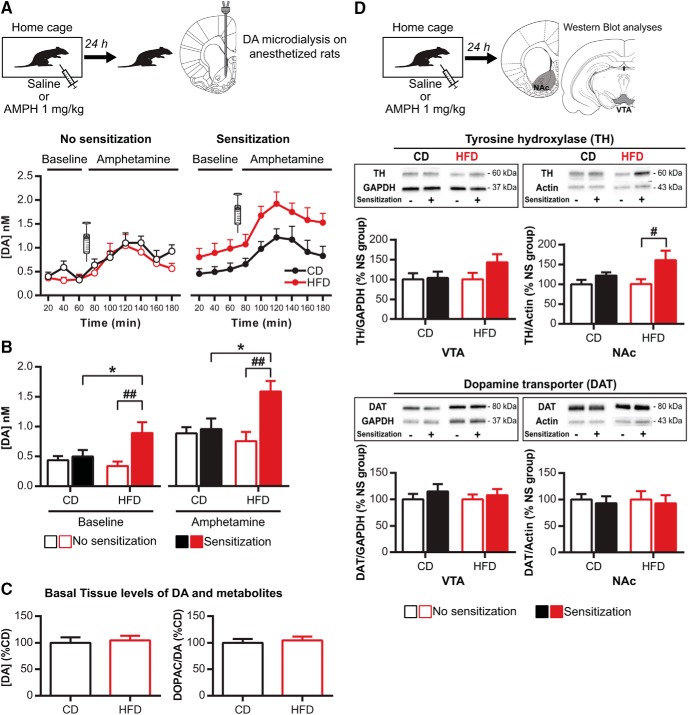
Amphetamine sensitization increases DA release and TH expression in the NAc after pHFD. ***A***, pHFD rats showed an increase in NAc DA release after amphetamine sensitization but not before (no sensitization CD *n* = 6 and pHFD *n* = 9; sensitization CD *n* = 9 and pHFD *n* = 8). Syringes represent the time of amphetamine injection. ***B***, Amphetamine sensitization increased NAc DA release only in pHFD group at both basal state and in response to a second injection of amphetamine. ***C***, Basal tissue levels of DA (left) and DOPAC-to-DA ratio (right) in the NAc was not altered by pHFD in nonsensitized rats (data are expressed in % of CD; CD *n* = 10 and pHFD *n* = 17). ***D***, Amphetamine sensitization increased expression of TH in the NAc and the VTA but did not affect DAT expression (data are expressed in % of respective nonsensitized group; no sensitization CD *n* = 11 and pHFD *n* = 10; sensitization CD *n* = 11 and pHFD *n* = 11). Data are expressed as mean + SEM. *p < 0.05 diet effect; #*p* < 0.05, ##*p* < 0.01 sensitization effect.

The activity of VTA DA cells is directly related to DA release in the NAc (Floresco et al., 2003) which is a central structure for behavioral sensitization processes (Vanderschuren and Kalivas, 2000; Ikemoto, 2002). In accordance with locomotor activity (Fig. 1), nonsensitized CD and pHFD groups displayed similar basal and amphetamine-induced DA levels assessed by microdialysis (diet: *F*_(1,12)_ = 0.7, *p* = 0.4; time: *F*_(8,96)_ = 10.3, *p* < 0.001; interaction: *F*_(8,96)_ = 0.7, *p* = 0.7; Fig. 3*A*, left). This absence of diet effect was confirmed, at basal state, by similar DA concentration (*t*_(25)_ = 0.8, *p* = 0.4) and DOPAC/DA ratio (*t*_(25)_ = 0.05, *p* = 0.9) in NAc tissue (Fig. 3*C*) as well as similar NAc expression of the DAT and the rate-limiting enzyme in DA synthesis TH (Table 1).

Consistent with our behavioral results, amphetamine sensitization induced an increase in DA levels only in pHFD-exposed rats at both basal state and in response to a second amphetamine injection [diet: *F*_(1,15)_ = 6.7, *p* < 0.05; time: *F*_(8120)_ = 14.9, *p* < 0.001; interaction: *F*_(8120)_ = 0.7, *p* = 0.7 (Fig. 3*A*, right); diet: *F*_(1,27)_ = 1.7, *p* = 0.2; sensitization: *F*_(1,27)_ = 7.1, *p* < 0.05; Block: *F*_(1,27)_ = 39.9, *p* < 0.001; diet × sensitization: *F*_(1,27)_ = 4.7, *p* < 0.05; interaction diet × block: *F*_(1,27)_ = 0.2, *p* = 0.6; interaction sensitization × block: *F*_(1,27)_ = 0.6, *p* = 0.4; interaction diet × sensitization × block: *F*_(1,27)_ = 0.5, *p* = 0.5 (Fig. 3*B*)]. Consistently, this increase was associated, in the pHFD group, with a higher TH expression in the NAc (CD: +21%, *t*_(20)_ = 1.6, *p* = 0.1; HFD: +60%, *t*_(19)_ = 2.1, *p* < 0.05) and a trend in the VTA (CD: +4%, *t*_(20)_ = 0.2, *p* = 0.8; HFD: +42%, *t*_(19)_ = 2.2, *p* = 0.1) without changes in DAT expression (all *t* < 0.8, *p* > 0.4; Fig. 3*D*). These results show that pHFD intake enhances the effect of amphetamine sensitization on the DA mesolimbic system by increasing VTA activity and NAc DA release.

### pHFD increases postsynaptic cellular changes in the NAc after amphetamine sensitization

**Figure 4.**
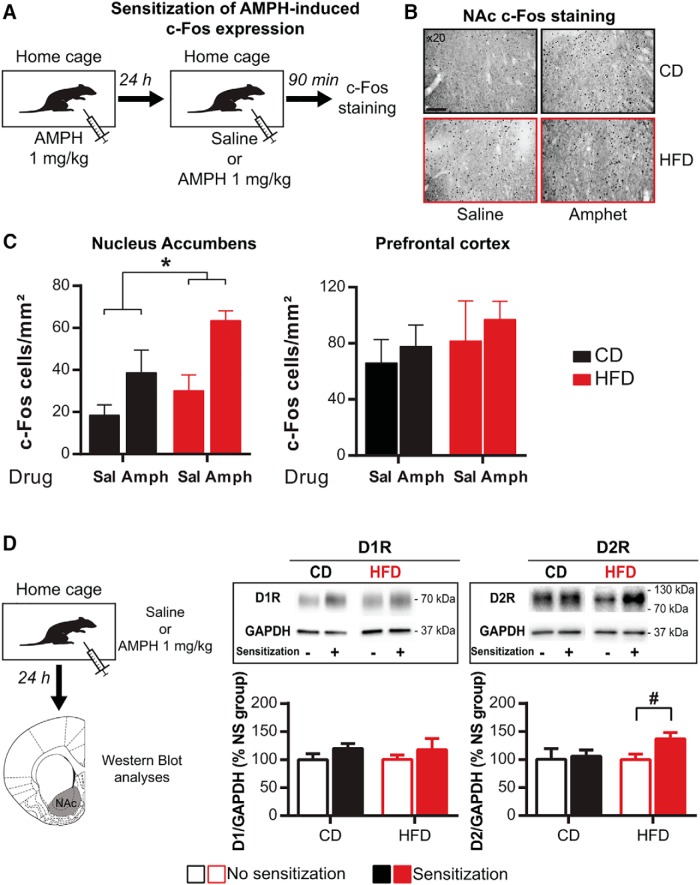
Amphetamine sensitization increases c-Fos expression and D2R expression in the NAc after pHFD. ***A***, Twenty-four hours after amphetamine injection, rats were perfused 90 min after an intraperitoneal injection of either saline (CD *n* = 5; pHFD *n* = 5) or amphetamine (CD *n* = 4; pHFD *n* = 5). ***B***, Representative pictures of c-Fos immunostaining in NAc for each experimental group at high magnification (20×). Scale bar, 100 µm. ***C***, Sensitized pHFD rats had more c-Fos levels in the NAc than CD animals, independently of saline or amphetamine injection but not in the prefrontal cortex (*n* = 4–5). ***D***, Amphetamine sensitization increased expression of D2R in the NAc (data are expressed in % of respective nonsensitized group; no sensitization CD *n* = 11 and pHFD *n* = 9; sensitization CD *n* = 11 and pHFD *n* = 10) but did not affect D1R expression. Data are expressed as mean + SEM. *p < 0.05 diet effect; #*p* < 0.01 sensitization effect.

Behavioral sensitization is mediated through the recruitment of NAc DA receptors leading to postsynaptic neuronal activity as revealed by the induction of c-Fos expression (Graybiel et al., 1990; Konradi et al., 1996; Valjent et al., 2005). To evaluate whether the increased response to amphetamine in pHFD rats also triggers postsynaptic changes in the NAc, we first measured c-Fos expression, 90 min after saline or amphetamine injection focusing on sensitized animals (Fig. 4*A*,*B*). As expected, amphetamine injection induced higher NAc c-Fos expression than saline injection in both groups. Strikingly, sensitized pHFD animals showed an overall significant higher level of NAc c-Fos than CD rats whatever the type of injection (diet: *F*_(1,15)_ = 6.8, *p* < 0.05; drug: *F*_(1,15)_ = 14.7, *p* < 0.01; interaction: *F*_(1,15)_ = 0.9, *p* = 0.4; Fig. 4*C*) demonstrating increased neuronal activity in the NAc of pHFD animals following amphetamine exposure. This pattern of c-Fos expression was not observed in the medial prefrontal cortex (all *F* < 0.8, *p* > 0.4; Fig. 4*C*), suggesting the mesoaccumbens DA pathway is more vulnerable to pHFD than the mesocortical DA pathway.

We next measured the expression of the DA receptors D1R and D2R in the NAc. Western blot analyses revealed no differences between CD and pHFD groups in basal condition (Table 1). A single injection of amphetamine 24 h before was sufficient to enhance the expression of D2R (+36%) compared with nonsensitized pHFD rats, which was not observed on CD animals (+5%; CD: *t*_(20)_ = 0.2, *p* = 0.8; HFD: *t*_(17)_ = 2.4, *p* < 0.05; Fig. 4*D*). No significant changes were observed for the expression of D1R for both CD and pHFD groups (all *t* < 1.4, *p* > 0.2).

## Discussion

The results of the present study reveal that chronic HFD from childhood to adulthood induces long-term alterations in the sensitivity of the DA mesolimbic pathway. Strikingly, using a short sensitization protocol (Valjent et al., 2005; Chinen et al., 2006; Valjent et al., 2010), we revealed that pHFD potentiates amphetamine-induced sensitization and adaptations of the VTA-NAc DA system. Previous studies have already shown a higher response to drug sensitization after obesogenic diet using a variety of diet conditions and psychostimulants (McGuire et al., 2011; Baladi et al., 2015; Robinson et al., 2015; Fordahl et al., 2016; Oginsky et al., 2016). However, we demonstrate here that a single drug-induced stimulation of the DA system in HFD-exposed animals is sufficient to induce behavioral and neurobiological adaptations, stressing the vulnerability of the DA mesolimbic system to HFD consumption.

We first report similar basal locomotor activity and response to a single amphetamine injection between CD and pHFD-exposed rats. Consistent with this behavioral pattern, pHFD did not affect DA cells activity, levels of DA and metabolites, DAT, TH, or DA receptors expression, suggesting a normal functioning of DA system before challenge, as supported by previous studies conducted in adult animals exposed to obesogenic diet (Décarie-Spain et al., 2015). By contrast to our results, however, several studies in human and animal models have reported that diet-induced obesity decreases the basal expression of striatal DA receptors D2R (Wang et al., 2009; Johnson and Kenny, 2010; Wang et al., 2011; Robinson et al., 2015; Friend et al., 2016). Such a discrepancy likely results from the moderate weight gain in HFD-fed rats in the present study as it was recently stressed that weight gain (influenced by the composition, the duration and the feeding pattern of the obesogenic diet) represents an important factor determining the impact of HFD intake on DA system (Décarie-Spain et al., 2015).

In the present study, since we aimed at investigating subtle changes in DA functioning, we used a two-injection protocol with low dose of amphetamine. Using this procedure, our results show that control rats did not increase their locomotor activity to the second amphetamine injection, a result which is consistent with previous research. Indeed, under such two-injection protocol, it has been repeatedly demonstrated that behavioral sensitization to psychostimulants depends on both the delay between drug exposures and the relative contextual similarity between the first and the second drug injection (Robinson and Berridge, 1993; Vanderschuren et al., 1999; Vanderschuren and Kalivas, 2000; Chinen et al., 2006; Steketee and Kalivas, 2011). The pattern of results obtained in pHFD animals was in contrast with the CD animals since they show an increased locomotor activity following the second drug administration, therefore demonstrating behavioral sensitization. Our results show that this behavioral effect might be related to changes in the sensitivity of the mesolimbic system as discussed below.

In the present study, pHFD-sensitized animals displayed an increased bursting activity of VTA DA cells. Behavioral sensitization involves complex interactions between glutamatergic and DA transmission (Graybiel et al., 1990; Konradi et al., 1996; Vanderschuren and Kalivas, 2000), even after a single exposure to drugs (Valjent et al., 2005; Valjent et al., 2010). Bursting activity of DA cells is highly controlled by glutamatergic excitatory inputs in the VTA (Georges and Aston-Jones, 2002; Floresco et al., 2003; Glangetas et al., 2015) that are quickly potentiated by drug exposure (Ungless et al., 2001). Since the consumption of palatable food already strengthens excitatory transmission on DA cells (Liu et al., 2016), it is possible that a single amphetamine injection is sufficient, in pHFD rats, to potentiate glutamatergic inputs on VTA DA cells, increasing their bursting activity. This increased activity of DA cells combined with the higher NAc TH expression in sensitized pHFD rats could likely participate to their higher NAc DA release that could, in turn, be responsible for the increased locomotor activity in response to amphetamine through the recruitment of postsynaptic DA receptors (Campbell et al., 1997; Heusner et al., 2003).

In striatal regions, postsynaptic D1R and D2R are mainly expressed by GABAergic medium spiny neurons (MSNs) segregated into two distinct output pathways, D1R-MSNs and D2R-MSNs (Le Moine and Bloch, 1995), which have functionally opposing effects on locomotion (Gerfen and Surmeier, 2011; Kravitz et al., 2012; Cui et al., 2013). Previous research in lean animals have demonstrated the critical involvement of D1R, but not D2R, in drug-induced striatal expression of c-Fos and locomotor sensitization (Graybiel et al., 1990; Konradi et al., 1996; Valjent et al., 2005; Valjent et al., 2010). Surprisingly, sensitized pHFD rats displayed up-regulation of NAc D2R, but not D1R. However, recent studies indicate that D2R-MSNs could also participate in locomotor sensitization. Inhibition of D2R-MSNs, mimicking DA action on D2R, does not change acute locomotor responses to amphetamine, but increases amphetamine sensitization (Ferguson et al., 2011). This effect seems to involve suppression of lateral inhibition exerted by D2R-MSNs on D1R-MSNs in the NAc (Dobbs et al., 2016). We therefore hypothesize that the higher DA release in sensitized pHFD rats induces greater NAc c-Fos levels through both the direct D1R stimulation as well as stronger disinhibition of D1R-MSNs due to D2R upregulation.

In summary, our study provides evidences that the chronic consumption of HFD during periadolescent period enhances the sensitivity of the mesolimbic DA system. Adolescence represents a key period of vulnerability to the effects of HFD on brain function (Noble and Kanoski, 2016; Reichelt, 2016). Interestingly, we recently showed that memory alterations induced by pHFD can be reversed by shifting HFD to CD (Boitard et al., 2016). However, protracted alterations of the mesolimbic DA system and reward-based processes were reported after the removal of adolescent HFD/high-sugar diet, suggesting different sensitivities of brain circuits to deleterious effects of palatable foods (Teegarden et al., 2009; Vendruscolo et al., 2010; Carlin et al., 2016; Naneix et al., 2016). Moreover, we previously demonstrated in rats that HFD consumption during adolescence enhanced basal levels of circulating leptin and induced protracted stress-induced release of glucocorticoids (Boitard et al., 2014; Boitard et al., 2015; Tantot et al., 2016). As leptin and glucocorticoids are important regulators of mesolimbic DA pathway and participate to amphetamine sensitization in lean animals (Fulton et al., 2006; Parnaudeau et al., 2014; Ferrario et al., 2016), it would be worthwhile to investigate the relationship between these hormonal changes and enhanced behavioral sensitization induced by pHFD. The enhanced sensitivity of the mesolimbic DA system induced by pHFD could impact reward processing. Whereas obesogenic diet consumption during adolescence decreases the motivation to work for rewards (Frazier et al., 2008; Vendruscolo et al., 2010; Naneix et al., 2016; Tantot et al., 2016), obesity is associated with specific enhancements of incentive properties of reward-related cues (Burger and Stice, 2011). The present study therefore highlights some neurobiological mechanisms which could support the increase incentive salience of food cues in obese patients. Given the increasing consumption of energy-rich foods in adolescents (Ogden et al., 2012), our results represent a step forward in the better understanding of the emergence of food-related disorders during development.

## Synthesis

Reviewing Editor: Carmen Sandi, Swiss Federal Institute of Technology Decisions are customarily a result of the Reviewing Editor and the peer reviewers coming together and discussing their recommendations until a consensus is reached. When revisions are invited, a fact-based synthesis statement explaining their decision and outlining what is needed to prepare a revision will be listed below. The following reviewer(s) agreed to reveal their identity: Mary Kay Lobo

The two reviewers and the editor assess this article very highly. It provides a notable advance in the field by demonstrating that early exposure to high fat diet consumption can alter the sensitivity of the dopamine system thus leading to altered reward-related processes. Previous studies for the effects of high-fat diet consumption during adolescence on DA system has been controversial, so this study adds a new piece of evidence in this field.

More precisely, the study describes the impact of periadolescent exposure to high-fat diet (HFD) on the mesolimbic dopaminergic system. The authors show that, although the baseline condition of mesolimbic DA pathway in rats with HFD is not apparently different from those who had normal chews, it is more vulnerable to amphetamine sensitization compared to controls. Specifically, HFD treatment increases locomotor sensitization to low doses of amphetamine. This increase is associated with higher VTA activity and dopamine release in the nucleus accumbens (NAc). Moreover, dopamine D2 receptors and c-Fos expression in the NAc of HFD-fed animals were increased after amphetamine injection.

The manuscript is well executed and well written with solid results to support the conclusion. The variety of techniques used offers a global view of the sensitization of the dopaminergic system after HFD exposure.

The reviewers have the following recommendations on minor issues to improve that can help improving the manuscript:

1. The authors show that sensitized HFD rats present higher locomotor sensitization compared to sensitized CD rats (Fig. 1C). It would be interesting to compare locomotion in sensitized HFD rats compared to non-sensitized HFD rats (shown in Fig. 1B). This could be briefly mentioned in the results section.

2. Figure 3A bottom left panel (No sensitization animals): to remain consistent with the labeling of the groups, open black and red circles could be used.

3. Figure 3C presents basal levels of DA and metabolites as % of CD rats. However in both cases, bars representing CD rats do not seem to reach 100%.

4. It would be interesting if the authors could add a few sentences discussing whether the effects of HFD presented in this study could be reversed by exposing HFD-fed rats to control diet.

5. Page 13, line 332, the authors mention aHFD instead of pHFD.

6. HFD was given to the period much longer than adolescence. Postnatal day (PD)21 when HFD started is definitely childhood, and it is stated in the method that HFD was given for 3 months, by which animals had reached way older than young adulthood. In addition, if HFD was given until the day before experiments, wasn't there any acute influence of HFD consumption?

7. Please provide a total number of animals used for all experiments in this research. In addition, please also provide exact number of animals used for each experiment. In figure legends, these are stated as n=xx-xx, which are too unclear to interpret the results. In addition, Probably, there were quite substantial number of animals not included in the final data, especially for those used in electrophysiology. How many animals were rejected due to mislocation of electrodes?

8. In relation to the above issue, how were electrophysiological recording sites confirmed histologically?

9. In Western blot, what is the rationale that GAPDH was used as control in the VTA, whereas actin was used as control in the NAc?

10. It looks like the Western blot band for D2R in HFD without sensitization shown in the figure 5D is weaker than any other conditions. Why?

11. Page 5, Line 92-93: Table 1 mentioned here does not match with Table 1 inserted in the manuscript. I guess it is Table 1 in Tantot et al., 2016 paper.
